# Supplementary material for: Normal tissue architecture determines the evolutionary course of cancer
Source: Nat Commun. 2021 Apr 6;12:2060. doi: 10.1038/s41467-021-22123-1 (PMC8024392; doi:10.1038/s41467-021-22123-1)
Supplement: Supplementary file 1 — Supplementary Information [file 41467_2021_22123_MOESM1_ESM.pdf]

Normal tissue architecture determines the evolutionary course of cancer

Jeffrey West[1,*], Ryan O. Schenck[1,2], Chandler Gatenbee[1], Mark Robertson-Tessi[1], and Alexander R. A. Anderson[1,*]

[1]Integrated Mathematical Oncology Department, H. Lee Moffitt Cancer Center & Research Institute, 12902 Magnolia Drive, SRB 4 Rm 24000H Tampa, Florida, 33612
[2]Wellcome Centre for Human Genetics, University of Oxford, Oxford, OX37BN, UK
[*]Corresponding authors: jeffrey.west@moffitt.org; Alexander.Anderson@moffitt.org

Supplementary Figures

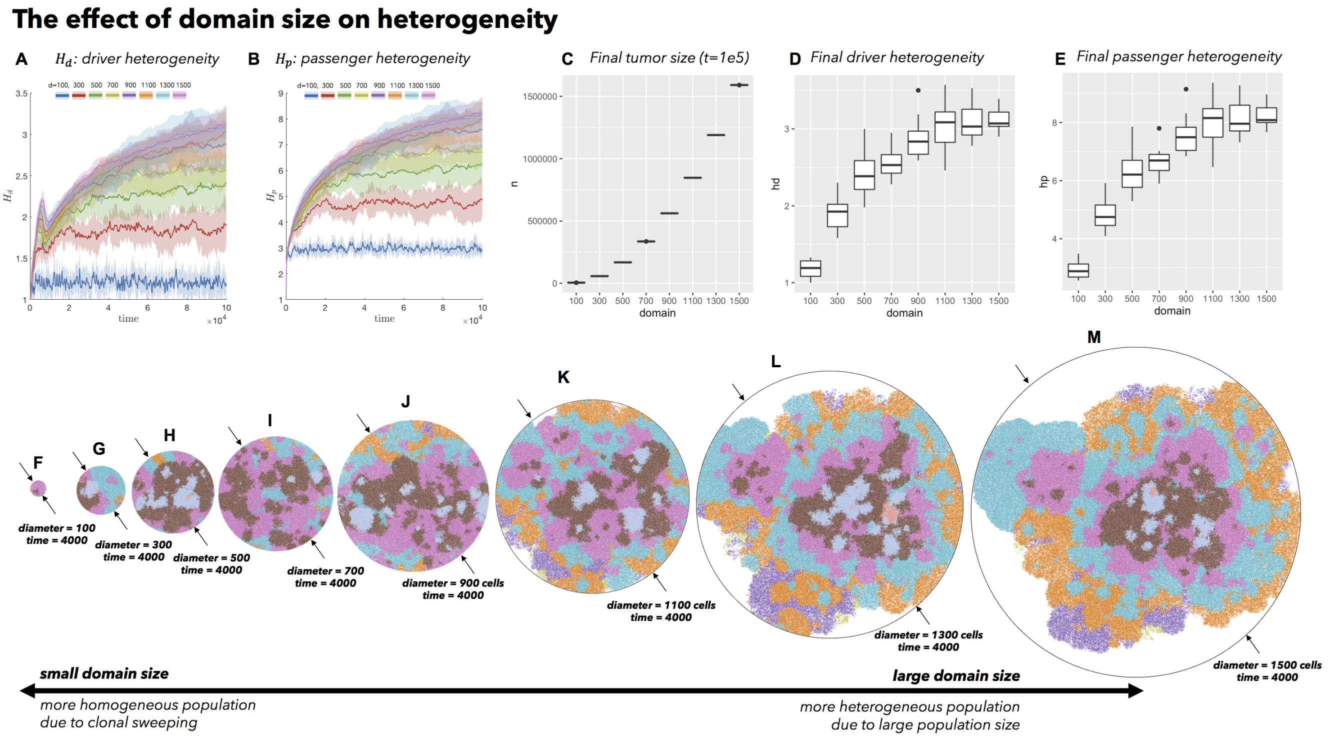

Figure S1. The effect of domain size on heterogeneity (A) Simulations with identical parameters as figure 1, top row, were performed for a larger range of domain sizes. (A,B) Driver and passenger heterogeneity increase with larger domain sizes. Solid lines indicate mean value, bands indicate ± 1 standard deviation for $N = 10$ simulations. (C,D,E) Box and whisker plots (showing median, 25/75 percentiles, and smallest/largest values within 1.5 times the interquartile range above/below quartiles) for $N = 10$ simulations for each domain size. (C) Final tumor size ($t = 1e5$)for each circular domain diameter size. (D,E) Final driver and passenger heterogeneity ($t = 1e5$) for the simulations in A and B. Increased domain sizes result in higher heterogeneity. However, larger domains reach steady state of heterogeneity after longer timescales, causing a tapering off of heterogeneity for largest domains. (F-M) Snapshots of the simulations after 4000 generations.

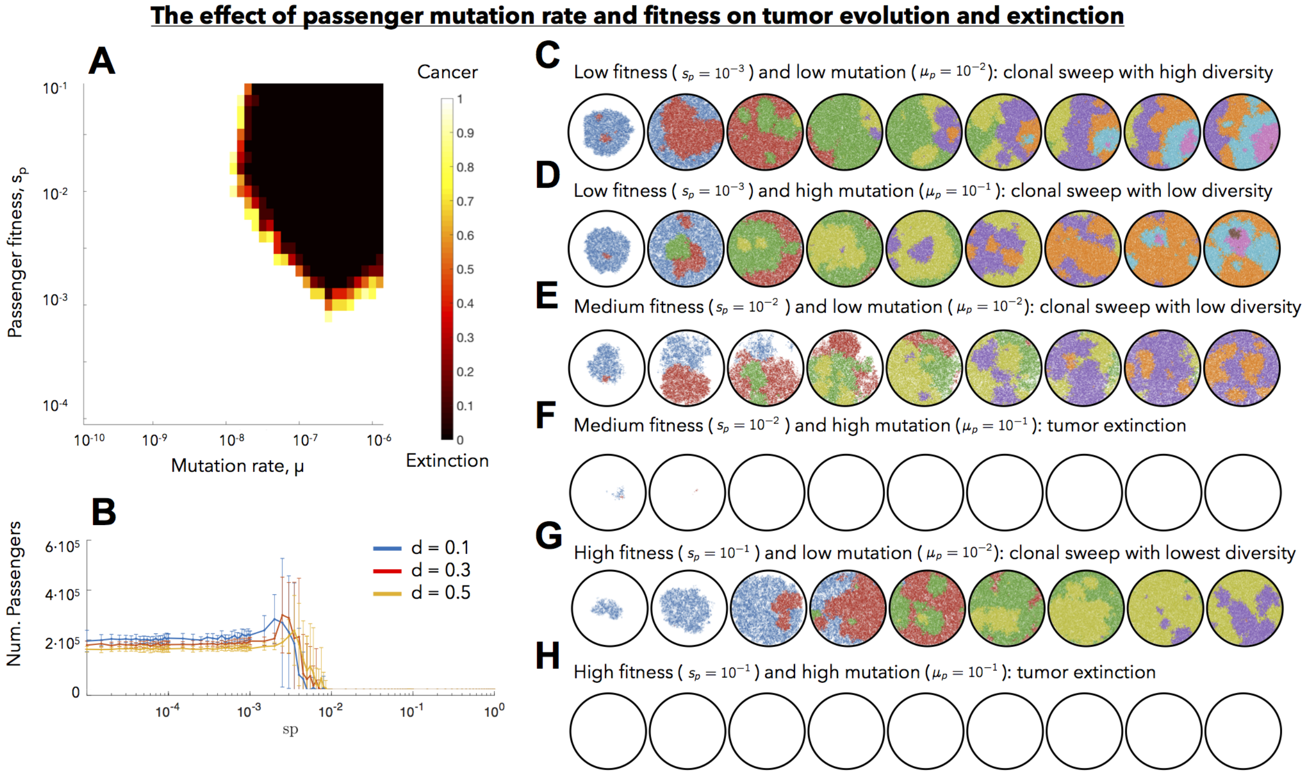

Figure S2. Cancer progression and extinction in passenger driver evolution. (A) Rate of cancer progression (simulations that do not go extinct after 10000 cell generations) and extinction depends on passenger fitness penalty, s_p and mutation rate, μ. 10 simulations shown for each parameter value. (B) Low and moderately deleterious passenger mutations can evade negative selection, for a variety of turnover (cell death, d) values. Lines show mean value; error bars shown ± 1 standard deviation for $N = 10$ simulations. (C) Low passenger fitness penalty and low mutation rate leads to clonal sweep with high diversity at each time point. (D) Low passenger fitness and high mutation rate leads to clonal sweep with lower diversity. (E) Moderate passenger fitness and low mutation rate similarly leads to clonal sweep with lower diversity. (F) Moderate passenger fitness with high mutation rate leads to extinction. (G) High passenger fitness with low mutation rate leads to clonal sweep with lowest diversity. (H) High passenger fitness with high mutation rate leads to tumor extinction.

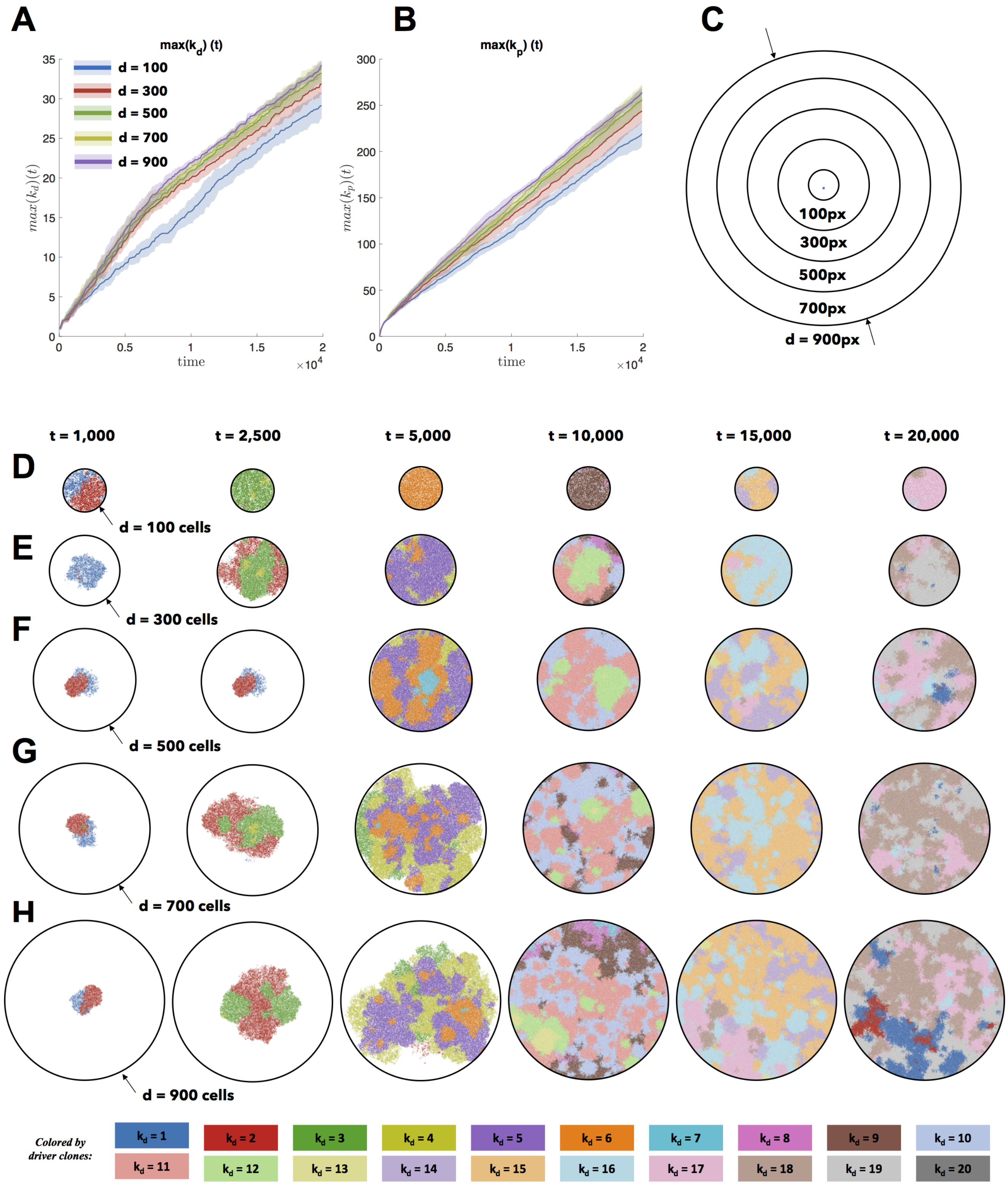

Figure S3. The effect of spatial competition on passenger hitch-hiking. (A,B) The maximum driver number in the population, $\max(k_d)$ and the maximum passenger, $\max(k_p)$ is shown for a range of size constraints. Solid lines indicate mean value, bands indicate ± 1 standard deviation for $N = 10$ simulations. Apart from the smallest domain size (D) of 100 cells, the maximum driver and passenger for each size constraint are relatively equivalent for each point in time. (C) Domain sizes, shown to scale. (D,E,F,G,H) As seen from the snapshots at each time point the population ranges from heterogeneous (large domains) to homogeneous (smaller domains).

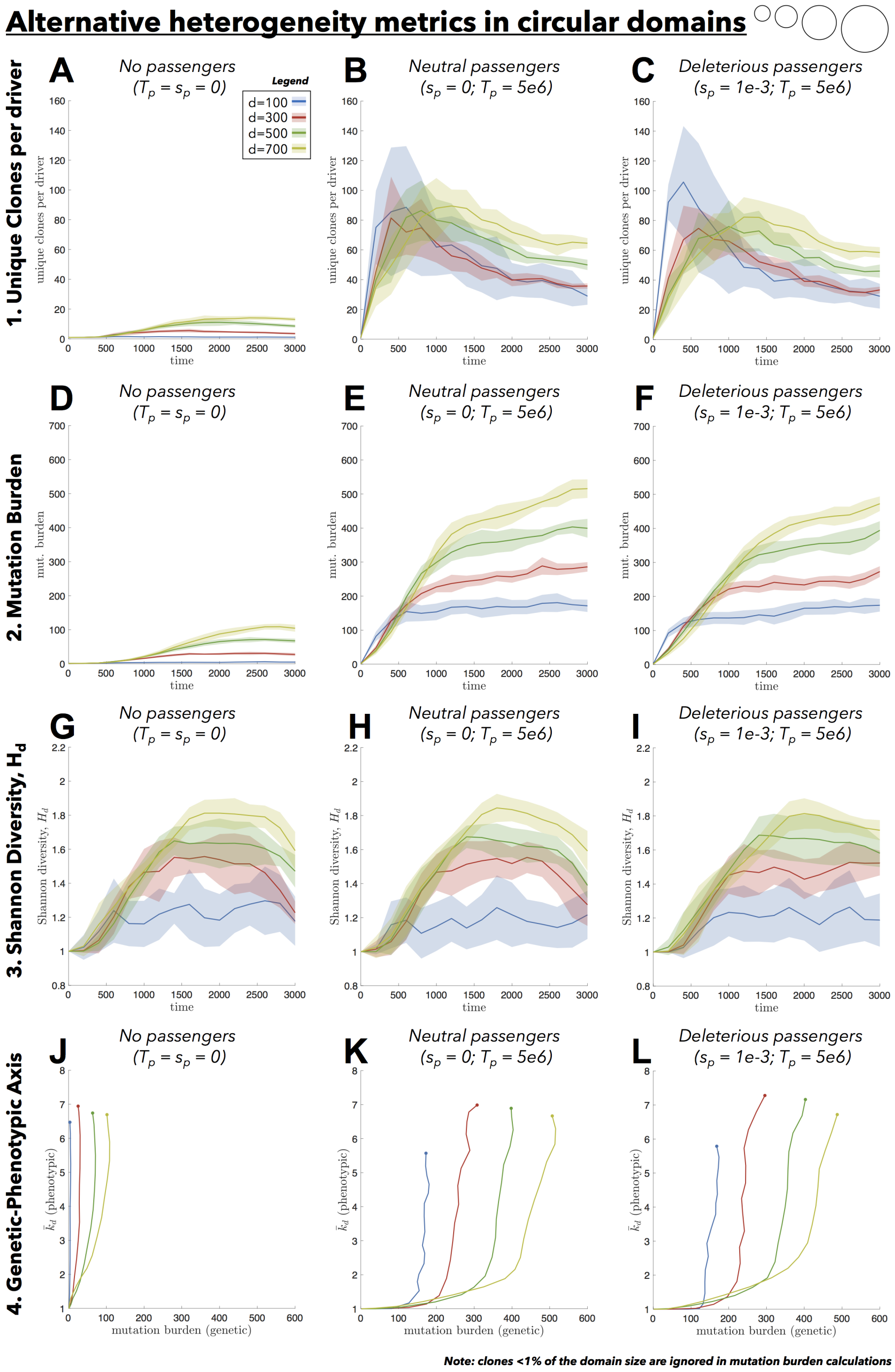

Figure S4. Alternative heterogeneity metrics in circular domains. This is an extension of the analysis done in figure 1 (top row): simulations on varied sizes of domains, ranging from 100 cells in diameter to 700 cells ($T_p = 5 \cdot 10^6$, $T_d = 700$, $s_d = 0.1$, $s_p = 0.01$). Small domain sizes increase the selection pressure, enabling fast sweeping and low diversity, across several alternative diversity metrics (rows), for neutral, nearly-neutral, or non-existent passenger mutations (columns). (A,B,C) show the total number of clones divided by the total number of distinct driver mutations. (D,E,F) show the mutation burden (i.e. the total number of clones). (G,H,I) show the Shannon diversity (see Methods). (J,K,L) show the trajectory through functional-genetic state space over time. The presence of passenger mutations substantially increases the number of clones (row 1 and row 2: compare first two columns). The greatest limiting factor on the total number of clones (the mutation burden) is the domain size, not negative selection due to deleterious passengers. Strong negative selection should be realized in a dramatic decrease in mutation burden, an effect not seen here. Solid lines indicate mean value, bands indicate ± 1 standard deviation for $N = 10$ simulations in a-i.

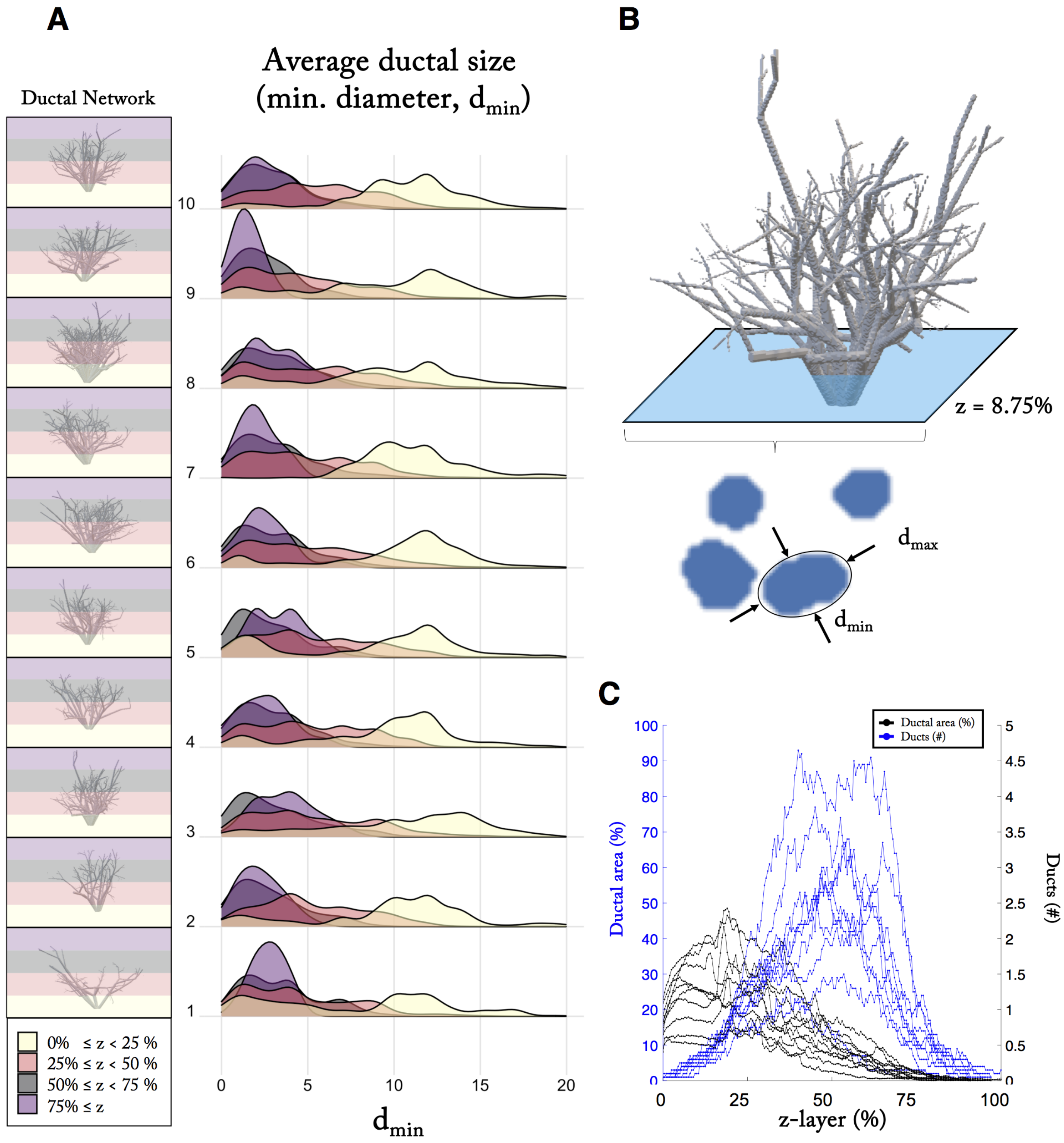

Figure S5. Quantification of spatial constraints imposed by breast ductal networks. (A) Data from ten anthropomorphic breast phantom ductal network reconstructions (20 stochastic simulations for each point of initiation in each ductal network). Distributions of average minimum diameter (shown in B) are shown for varied z (relative distance from ductal root). (B) For each two-dimensional slice, the number of ductal branches are counted and quantified using Python OpenCV. In order to minimize bias introduced when ductal branches run parallel to a given slice, an ellipse is fit (B, bottom) to each ductal branch. (C) Ductal area (percentage) and number of ducts is shown as a function of z (relative distance from ductal root). In general, there are fewer, larger ducts near the root of the ductal network and many, smaller ducts as z-layer increases.

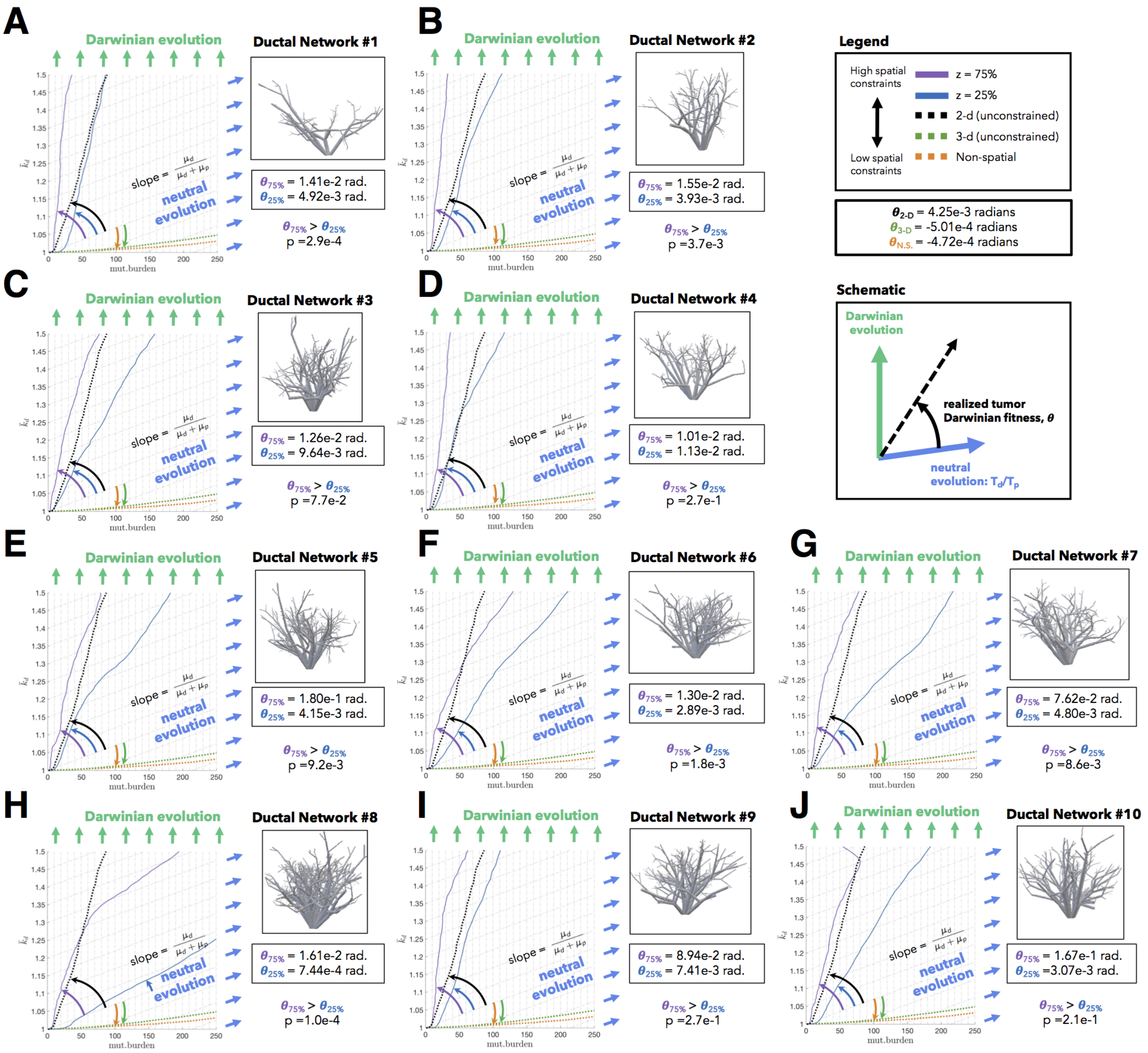

Figure S6. Statistical significance of realized tumor fitness, θ with varied initial spatial constraints. Data from ten anthropomorphic breast phantom ductal network reconstructions (shown in S5) constrain tumor growth in simulations. Trajectories are shown initiated in high spatially constrained regions of the ductal network ($z = 75\%$) and low spatially constrained regions ($z = 25\%$). Angles and p-values (one-tailed t-test) are reported in (A) through (J). These important conclusions are lost when considering identical parameterizations of a non-spatial (orange dashed line), two- (black dashed line), or three-dimensional (green dashed line) model.

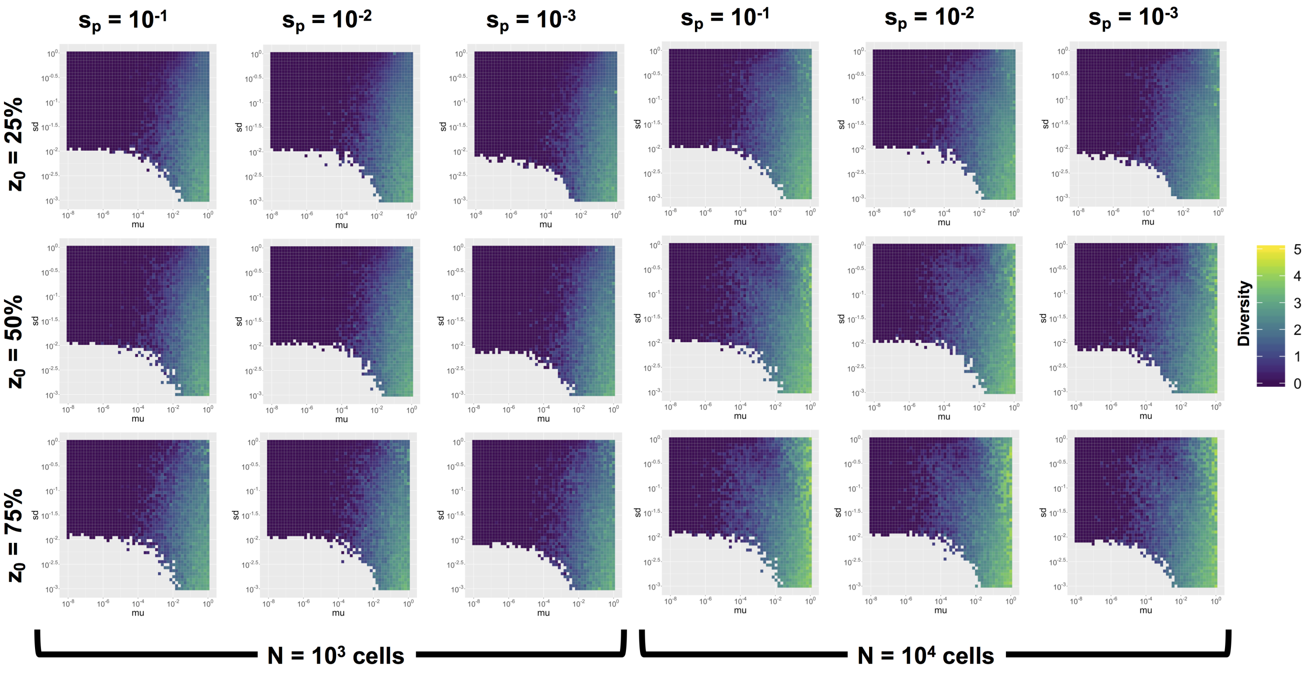

Figure S7. Model parameterization of clonal heterogeneity: Simulations were performed over a range of parameter values for driver mutation rates ($\mu_d \in [10^{-8}, 0]$; x-axis) and driver fitness ($s_d \in [10^{-3}, 1]$; y-axis). This parameter space is binned into 50 by 50 grid points, where each grid point is colored according to the classical definition of Shannon entropy ($-\sum_i (p_i \log p_i)$; see Methods) at the end of simulation. This is repeated for simulations stopped upon reaching $N = 10^3$ cells (left) and $N = 10^4$ cells (right). The effect of initial spatial location ($z \in [25, 50, 75]\%$; rows) passenger fitness ($s_p \in [10^{-3}, 10^{-2}, 10^{-1}]$). Shown for $\mu_p = 10^{-2}$. The trend of diversity remains similar in all cases with high mutation rate resulting in high heterogeneity.

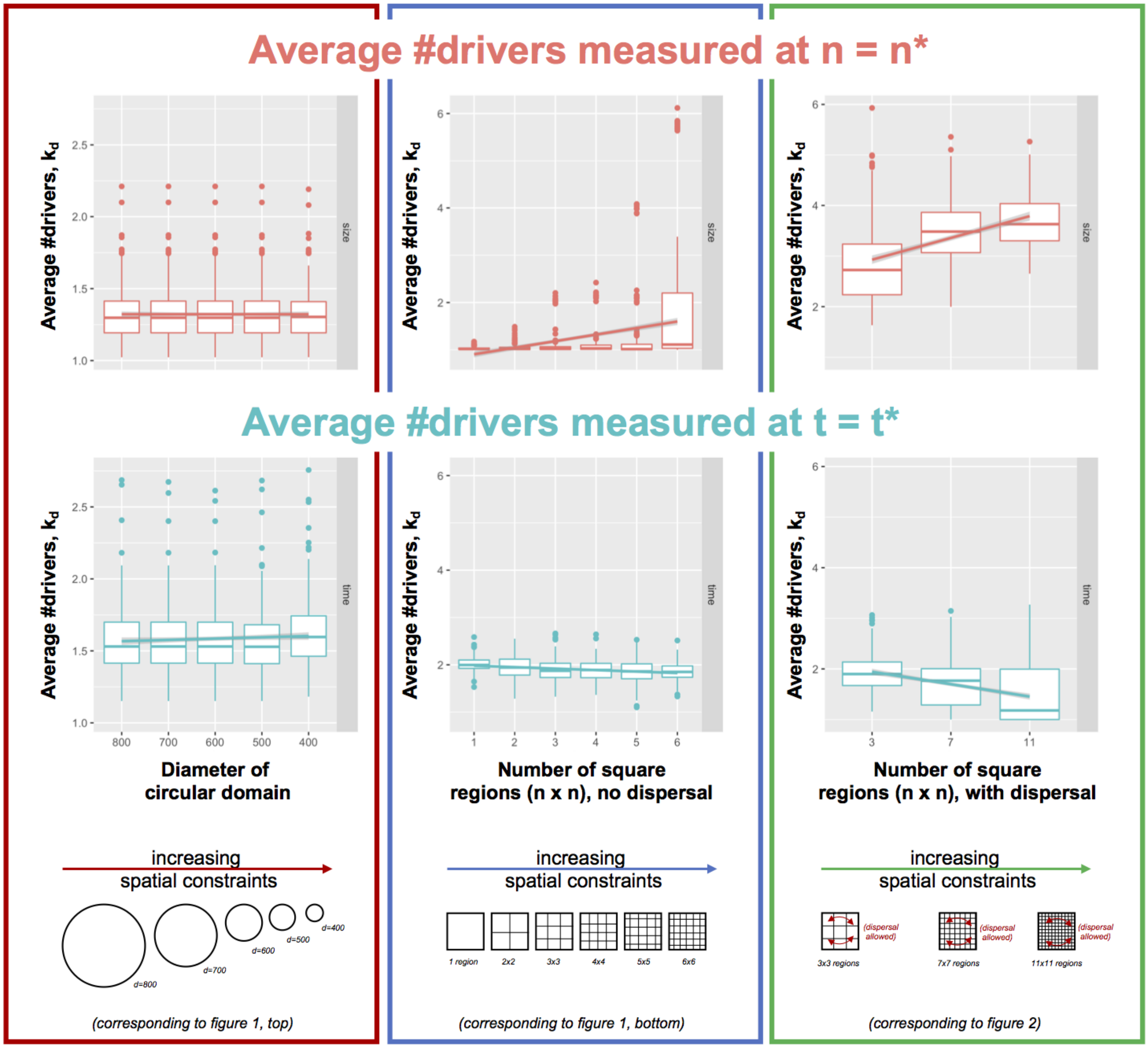

Figure S8. Direct comparison of spatial configurations: 200 simulations were performed for each spatial configuration: circular domains of varied size (red box), square domains of equal size (no dispersal; blue box), and square domains (with dispersal; green box). Here, the parameters and initial conditions are identical ($T_p = 1 \cdot 10^6$, $T_d = 700$, $s_d = 0.1$, $s_p = 10^{-3}$, $\mu = 10^{-8}$) Top row: average drivers measured at $n* = 62,500$ cells. increased spatial constraints (left-to-right) has little effect on the average drivers, $\bar{k}_d$,for domains without dispersal (red, blue). Drivers are increased simulations with dispersal (relative to the no dispersal baseline in blue) for all, and continue to increase with harsh spatial constraints (positive slope). Bottom row: average drivers measured at $t* = 1000$ generations. Again, increased spatial constraints has little effect on drivers without dispersal (red, blue), and shows a negative correlation with dispersal. Box and whisker plots show median, 25/75 percentiles, and smallest/largest values within 1.5 times the interquartile range above/below quartiles) for $N = 200$ simulations for each domain size.

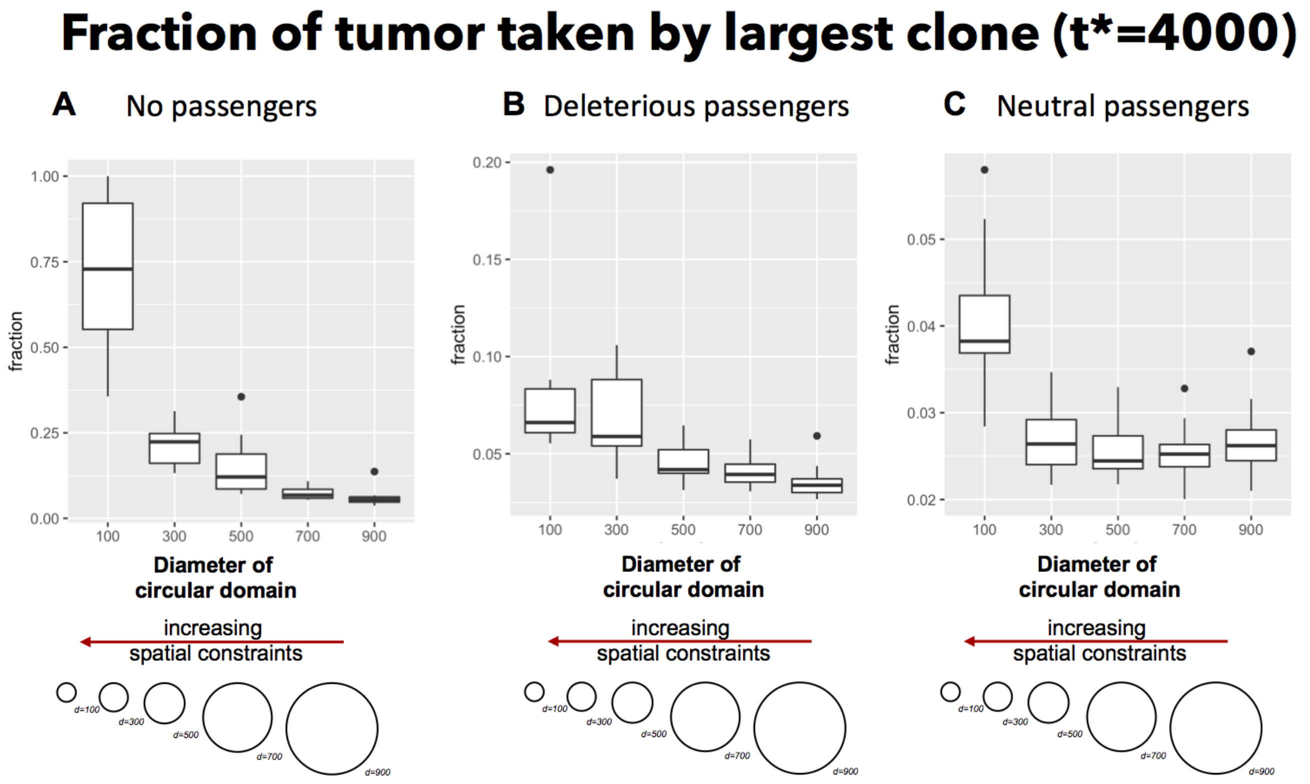

Figure S9. Fraction of tumor take by largest clone in varied domain size: Simulations with identical parameters as figure 1, top row, were performed for no passengers (A; $\mu_P = 0$), deleterious passengers (B; $s_p = 0.01$), and neutral passengers (C; $s_p = 0$). (A) In smaller domains (without passengers) the largest clone counts for the majority of cells within the tumor. The fraction of largest clone decreases as domain size increases. (B,C) This qualitative trend continues for deleterious and neutral passengers. The largest clone is smaller with passengers included because the number of clones increases. See companion Supplemental Video V6. (Parameters: $T_p = 5 \cdot 10^6$, $T_d = 700$, $s_d = 0.1$, $s_p = 0.01$, $\mu = 10^{-8}$) Box and whisker plots show median, 25/75 percentiles, and smallest/largest values within 1.5 times the interquartile range above/below quartiles) for $N = 10$ simulations for each domain size.
