# Supplementary material for: Normal tissue architecture determines the evolutionary course of cancer
Source: Nat Commun. 2021 Apr 6;12:2060. doi: 10.1038/s41467-021-22123-1 (PMC8024392; doi:10.1038/s41467-021-22123-1)
Supplement: Supplementary file 3 — Description of Additional Supplementary Files [file 41467_2021_22123_MOESM3_ESM.pdf]

Description of Additional Supplementary Files

File Name: Supplementary Movie 1
Description: **The effect of spatial constraints on heterogeneity (video):** Identical simulations and parameterization as figure 1 (top row). Cells divide and die on a regular square lattice. A cell selected for birth can divide only into an empty grid location and may accrue passenger or driver mutations. Simulations on varied sizes of domains, ranging from 100 cells in diameter to 900 cells, seeded with 100 cells at time zero (kd = 1, kp = 0) at time zero (Tp = 5x106, Td = 700, sd = 0.1, sp = 0.01). Cells are color-coded by number of drivers (top row) and number of passengers (bottom row).

File Name: Supplementary Movie 2
Description: **The effect of spatial constraints on heterogeneity (video):** Identical simulations and parameterization as figure 1 (bottom row). Cells divide and die on a regular square lattice. A cell selected for birth can divide only into an empty grid location and may accrue passenger or driver mutations. Identical domain size of 500 by 500 cells is seeded with one-third of the domain filled (kd = 1, kp = 0) and segregated into varied number of non-interacting regions (Tp = 106, Td = 700, sd = 0.1, sp = 0.01, μ = 10-8). Cells are color-coded by number of drivers (top row) and number of passengers (bottom row).

File Name: Supplementary Movie 3
Description: **Spatial segregation with cell dispersal accelerates evolution (video):** Identical simulations and parameterization as figure 2. Simulations seeded with 100 cells (kd = 1, kp = 0) are allowed to disperse between segregated regions at a low rate (0.01; left column) or high rate (0.1; right column) for varied number of segregated regions, as shown. A Muller plot of tumor evolution represents genotypes color-coded by driver (kd) value. The horizontal axis is time (cell generations), with height corresponding to genotype frequency. Descendant genotypes are shown emerging from inside their parents. Simulations repeated for 7 by 7 regions, and 11 by 11 regions. Parameters: Tp = 5x106, Td = 700, sd = 0.1, sp = 10-3, μ = 10-8.

File Name: Supplementary Movie 4
Description: **Three dimensional model of tumor evolution constrained by ductal network structure (video):** Identical simulations and parameterization as figure 3. Realistic three-dimensional topology of breast ductal networks (reconstructed with data from anthropomorphic breast phantoms) provides full three-dimensional maps to seed and constrain tumor evolution simulations. Tumor evolution is shown for varied initial conditions (Tp = 106, Td = 700, sd = 0.1, sp = 0.1, μ = 10-8). Simulations closer to the ductal root (top) in larger, less constrained branches are characterized by more consistently neutral evolution and constant acquisition of new clones. Simulations further from the ductal root (bottom) in smaller, more constrained branches are characterized by clonal sweeping early, but neutral evolution at later times. Each simulation is run for 3000 cell generations.

File Name: Supplementary Movie 5
Description: **Realistic three-dimensional topology of breast ductal networks (video**): Left: realistic three-dimensional topology of breast ductal networks, reconstructed with data from anthropomorphic breast phantoms provides full three-dimensional maps to seed and constrain tumor evolution simulations. This provides a topology of a continuously connected series of

progressively smaller ductal branches. Middle: slices in the z-dimension show fewer, larger ducts for low z-values and many, smaller ducts for high z-values. Right: static images of z-dimension slices.

File Name: Supplementary Movie 6
Description: **The effect of domain size on two largest clones (video):** Simulations with identical parameters as figure 1, top row, were performed for no passengers (μp=0; see figure S9A). The largest clone at each point in time is colored dark blue, with second largest colored light blue. All other cells are colored dark gray. Visually, smaller domains (left) consist of these two dominant clones, while the fraction of largest clone is smaller for large domains. See the stacked bar chart to the right of each domain for a more relative quantification of the sizes.
